# Single-stage debridement and spinal fusion using PEEK cages through a posterior approach for eradication of lumbar pyogenic spondylodiscitis: a safe treatment strategy for a detrimental condition

**DOI:** 10.1186/s13037-015-0083-4

**Published:** 2015-11-10

**Authors:** Sven K. Tschöke, Holger Fuchs, Oliver Schmidt, Jens Gulow, Nicolas H. von der Hoeh, Christoph-E. Heyde

**Affiliations:** Department of Spine Surgery, HELIOS Park Hospital Leipzig, Leipzig, Germany; Department of Spine Surgery, University Hospital Leipzig, Leipzig, Germany

**Keywords:** Pyogenic spondylodiscitis, Lumbar spine, Spondylodesis, PEEK cage, Surgical therapy

## Abstract

**Background:**

Pyogenic infections of the lumbar spine are a rare but critical pathology, yet with considerably high mortality rates. In cases indicating surgical therapy, the implantation of titanium cages or autologous bone grafts represent today's gold standard. Although non-metallic implants such as poly-ether-ether-ketone (PEEK) have proven to be advantageous in diverse degenerative conditions, their saftey and practicability in lumbar spine infection remains questionable. Moreover, the efficacy of a single-step radical debridement of the infected disc space with subsequent fusion from a strictly posterior approach continues to be an issue of debate. We therefore sought to evaluate the feasibility, clinical and radiological outcome of a single-step TLIF procedure using oblique PEEK cages in the surgical management of patients with lumbar pyogenic spondylodiscitis.

**Methods:**

From January 2009 through December 2013, all patients meeting the indication for surgical treatment of lumbar pyogenic spondylodiscitis were included. Patients demonstrating intact cortical bone on preoperative CT received a single-step radical debridement of the infected intervertebral disc space, posterior screw-and-rod instrumentation and implantation of an oblique PEEK cage using the TLIF technique. Oral antibiotics were continued for 12 weeks postoperatively and clinical and radiological results recorded throughout a minimum 1-year clinical follow-up.

**Results:**

A total of 104 patients were admitted to receive surgical therapy for lumbar pyogenic spondylodiscitis. Within this patient population, 18 patients met the diagnostic criteria to receive the implantation of an oblique PEEK cage. Pathogens were detected in 13 cases with Staph. aureus being the predominant causative organism. All patients were discharged to recover in their domestic environment. Throughout the first year of clinical and radiological follow-up and beyond, none of the 18 patients demonstrated any signs of residual neurologic deficits or recurrent infection. Furthermore, two-plane conventional X-rays showed no significant implant subsidence or failure at any of the given time-points in up to 5 years postoperatively.

**Conclusions:**

In patients meeting the criteria for surgical treatment of lumbar pyogenic spondylodiscitis, the implantation of PEEK cages using a single-step TLIF approach is a safe and feasible procedure. Based on our experience, the concern of a recurrent infection when implanting non-metallic cages may be refuted in carefully selected patients.

## Introduction

Pyogenic infections of the spine remain a seldom entity, yet associated with a high mortality of up to 17 % [1, 2]. Mild cases of spondylitis and/or spondylodiscitis without significant osseous destruction and consecutive instability may be successfully managed by conservative treatment, primarily antibiotic therapy and bracing. However, in more advanced states, particularly those with sepsis, impending or current deformity, significant instability and/or neurological compromise, a surgical treatment is mandatory. Thus, posterior pedicle screw-and-rod instrumentation, along with decompression of the spinal canal, radical debridement of the infected disc and intervertebral fusion using either titanium cages or autologous bone graft are considered today’s gold standard [3–6]. Although tricortical bone strut grafting for intervertebral fusion is widely accepted, the frequent incidence of a coherent donor site morbidity (e.g. persistent pain or fracture) remains a serious postoperative management problem [7]. In addition, the use of titanium cages is a reliable construct with regard to fusion rates, secondary kyphotic deformity and other primarily graft-associated complications (e.g. subsidence, fracture and necrosis/osteolysis) [4, 5, 8–11]. Moreover, recent advances in the development and design of titanium implants have enabled spine surgeons to complete both the requisite debridement of the infected disc and correction of deformity from a single posterior approach. In patients, where degenerative deformity (e.g. spondylolisthesis, lumbar scoliosis) and/or multiple comorbidities are present, limiting the surgical procedure to a single approach and event may significantly influence its respective result.

In contrast to the wide-spread use of poly-ether-ether-ketone (PEEK) as a biocompatible alternative to metal implants in various degenerative spinal disorders, its implementation within the primary surgical treatment of pyogenic spondylodiscitis remains an issue of debate. Although initial observations in recent small cohort studies including patients with cervical and or thoracolumbar/lumbar spondylodiscitis [11–13] have shown promising results, the implantation of PEEK as a synthetic material into a formerly infected site has not yet obtained general acceptance. This study demonstrates our initial results from a non-randomized controlled study including 18 patients with lumbar pyogenic spondylodiscitis treated by a standard TLIF-technique using oblique PEEK-cages with posterior screw-and-rod instrumentation. Furthermore, we discuss these results in the context of the most recent literature and our own experience with titanium cages.

## Material and Methods

### Patients

Patients presenting with pyogenic spondylodiscitis requiring surgical treatment were prospectively enrolled into the study upon admission to the Departments of Orthopaedic Surgery of the University Hospital Leipzig and the Department of Spine Surgery at the HELIOS Park Hospital Leipzig from January 2009 through December 2013. Indication for surgery included either failure of conservative treatment with image verified disease progression, secondary instability due to critical osseous lesions of the bony endplates or cortical bone, secondary lumbar kyphosis, immobilizing pain and/or neurological impairment. Patients demonstrating intact cortical bone of the adjacent vertebral endplates on CT were selected to receive an oblique PEEK cage as described below. All other patients received a standard titanium cage using the same surgical approach. Patients presenting with an infectious pathology of more than two respective segments were excluded from this study.

Laboratory chemical parameters included a routine blood count, C-reactive protein (CRP) serum levels and, in cases of systemic inflammatory response syndrome (SIRS) or sepsis, plasma levels of procalcitonin (PCT). The neurologic state was evaluated according to the American Spinal Injury Association (ASIA) impairment scale. In patients presenting a state of critical illness prior to surgery, multiple organ dysfunction was rated according to the cumulative component. The Multiple Organ Dysfunction Score (MODs) was applied as described by Marshall and colleagues (cardiovascular, hematologic, hepatic, neurologic, pulmonary, and renal) [14]. SIRS and/or sepsis were defined as previously outlined by the International Sepsis Definition Conference [15]. Written and informed consent was obtained prior to clinical documentation and surgery. In cases where written and informed consent was unfeasible due to the nature of the patient’s clinical state and/or course of treatment, the consent was obtained from the closest dependent, respectively.

### Imaging

Following the mandatory clinical evaluation, all patients were diagnosed by two-plane radiographic imaging of the respective spinal region and subsequent magnetic resonance imaging (MRI) of the complete spine with contrast medium. MRI was performed on 1.5-T imagers (Infera, Philips) using a surface coil or spine coil. Axial and sagittal T1-weighted MR images (TR range/TE range, 350–650/11–30) and fast spin-echo or turbo spin-echo T2-weighted images (3,000–4,000/76–108) were obtained. In addition, axial and sagittal fat-suppressed T1-weighted images (350–800/11–30) were obtained after IV infusion of 0.1 mmol/kg of gadoteric acid (Dotarem®, Guerbet, France). Presence or absence of individual imaging criteria was evaluated to make an overall assessment of the type of spondylodiscitis, respectively. The signal intensity in the marrow of abnormal vertebrae was considered hypointense, isointense, or hyperintense by comparison with the signal intensity of normal vertebrae in the same patient on T1- and T2-weighted images. Based on the indication for surgery, patients were subject to an additional 1 mm thin-film computer tomography (CT) scan of the affected vertebral level to evaluate the integrity of the bony structures. Significant osseous lesions of the cortical bone were considered inapt for the implantation of an oblique PEEK cage due to the risk of deficient bridging stability of the implant.

### Surgery

In all cases meeting the above-mentioned criteria for surgery, the surgical strategy included a single-stage posterior pedicle screw-and-rod instrumentation, followed by the radical resection of the affected intervertebral disc, bony debridement and intervertebral fusion.

The surgical approach was performed exclusively from posterior, thereby complementing the conventional open posterior pedicle screw-and-rod instrumentation with a radical resection of the infected disc and isochronal debridement of the intervertebral disc space and bony endplates. After removal of the infected tissue, the disc space was irrigated with an antiseptic solution (Lavasorb®, Fresenius Kabi AG, Bad Homburg, Germany), followed by normal saline. Subsequently, the intervertebral PEEK cage (MectaLIF PEEK oblique Cage, Medacta, Switzerland) was filled with a blend of gentamycin foam (25 cm^2^ Genta-Coll®, Resorba Medical GmbH, Nuernberg, Germany) and autologous bone graft and inserted oblique to bridge the complete diameter of the bony endplates in a transforaminal-lumbar-interbody-fusion (TLIF) technique. Each autologous bone graft was obtained from the respective facetectomy and laminotomy as part of the TLIF procedure. No additional iliac crest bone harvesting, allograft or bone substitute material was required. Thereafter, the wound was closed in a standard single-layer fashion, thus completing the surgical procedure.

### Postoperative treatment and clinical/radiographic follow-up

All patients continued antibiotic treatment for 12 weeks postoperatively. In cases where no pathogen was identified, broad-spectrum antibiotic regimens, primarily clindamycin, were applied. Radiographic (plain lateral and AP conventional radiographs) and clinical follow-up (C-reactive protein, leukocyte count) were performed 6 weeks, 3 months, 6 months and 1 year postoperatively. Thereafter, clinical and radiographic follow-up were continued on an anual basis. In addition, pain on a Visual Analogous Pain Scale (VAS) and the Oswestry Disability Index (ODI) were recorded. Postoperative bracing was not applied.

## Results

**Fig. 1:**
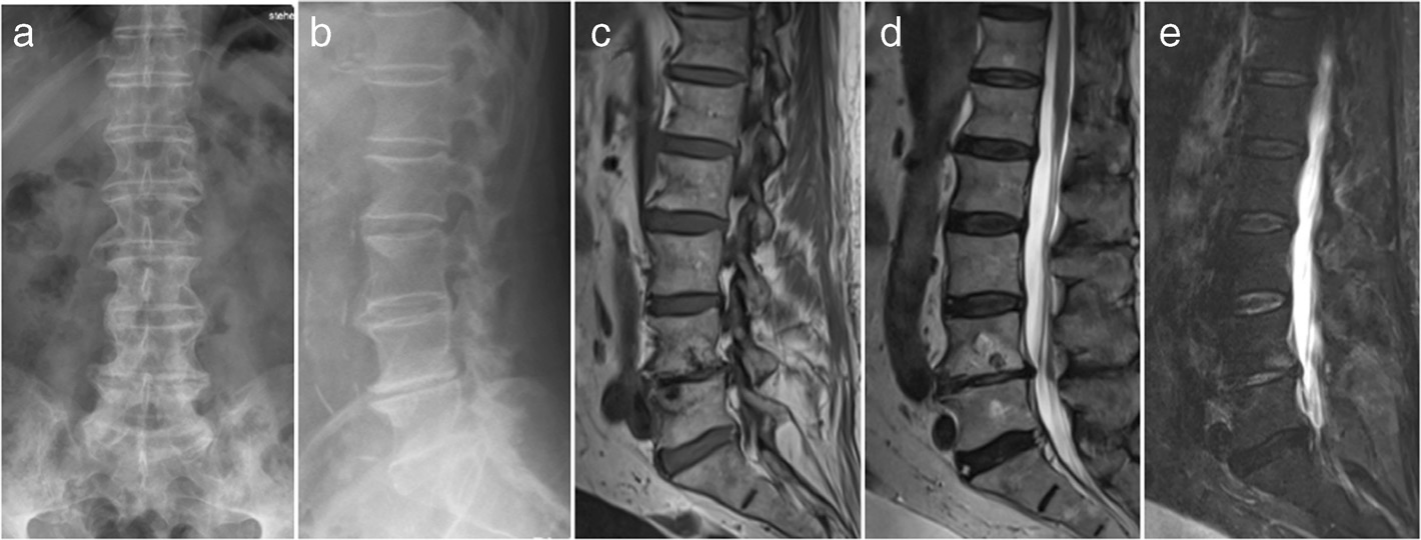
Examplary case of an 81 year old male patient presenting with immobilizing low back pain (VAS 8/10), fever and an elevated CRP serum level of 52 mg/l after 6 weeks of appropriate conservative therapy. Conventional X-ray images upon admission in an upright standing position a.p. (**a**) and lateral (**b**) Pre-operative sagittal MR T1-weighted (**c**) T2-weighted (**d**) and STIR (**e**) images demonstrating the bony endplate lesions at the L4/5 level

**Fig. 2:**
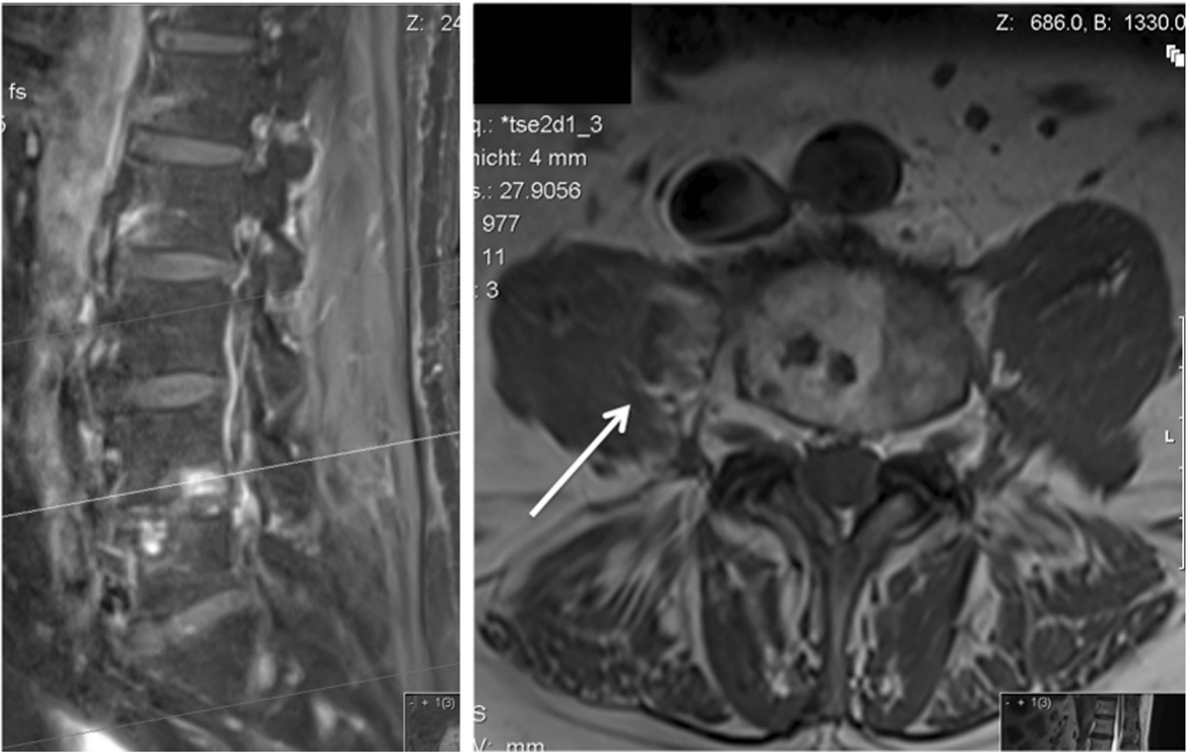
Sagittal (*left*) and axial (*right*) fat-suppressed T1-weighted MR images after IV infusion of 0.1 mmol/kg of gadoteric acid demonstrating an intramuscular abscess in the right psoas muscle at the L4/5 level (*white arrow*)

**Fig. 3:**
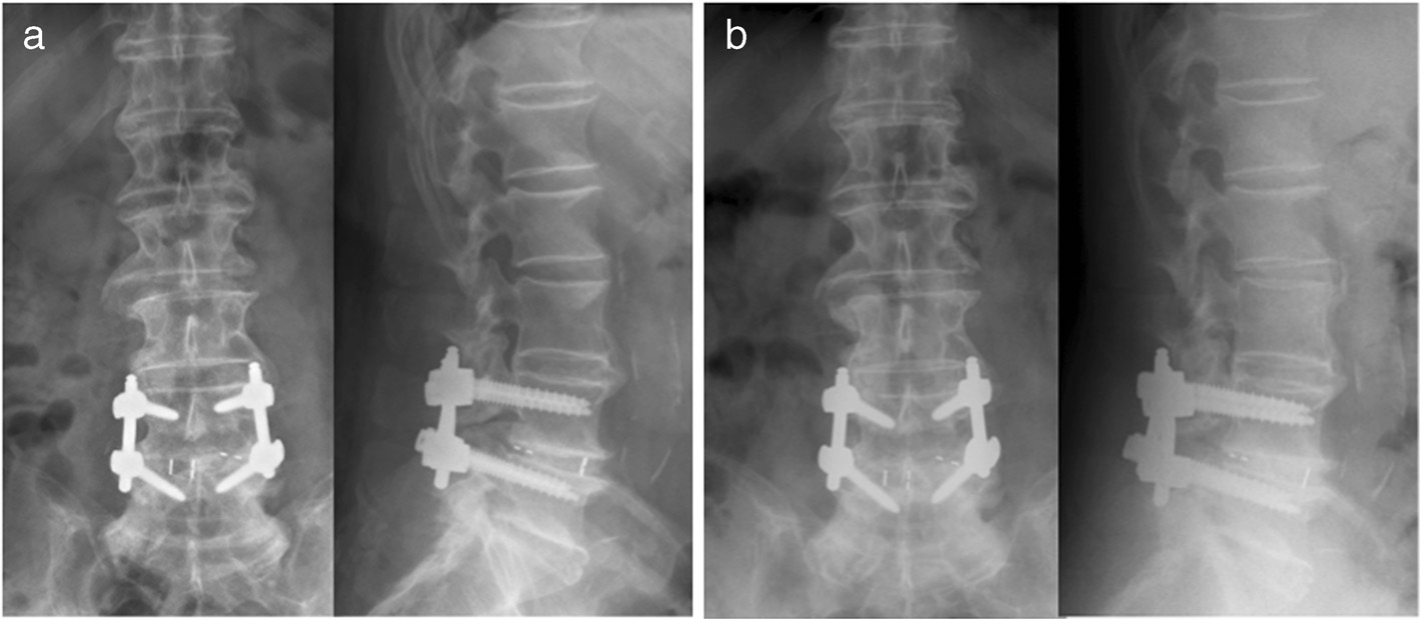
Post-operative a.p. and lateral conventional X-rays in an upright standing position at 3 months (**a**) and 1 year (**b**) postoperative follow-up

**Fig. 4:**
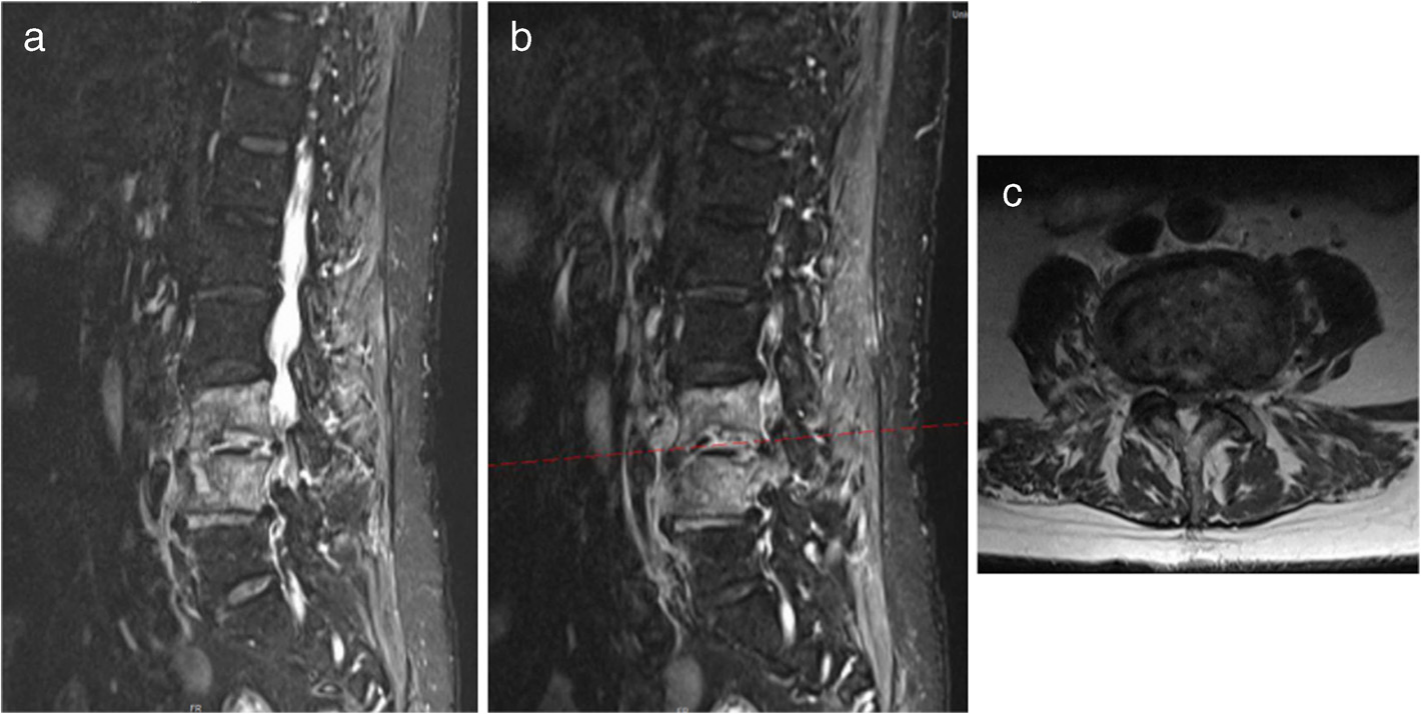
Examplary case of a 77 year old female patient presenting with immobilizing low back pain (VAS 10/10), instability, secondary kyphosis and an elevated CRP level of 119 mg/l. Pre-operative sagittal MR T2-weighted (**a**) and STIR (**b**) images demonstrate significant signal enhancements with arrosions of the bony endplates at the L3/4 level, along with a suspected infection of the L4/5 disc. The axial MR T2-weighted image of the L3/4 level (**c**) shows advanced degenerative changes of the facet joints with consecutive bilateral stenosis of the neuroforamen and spinal canal

**Fig. 5:**
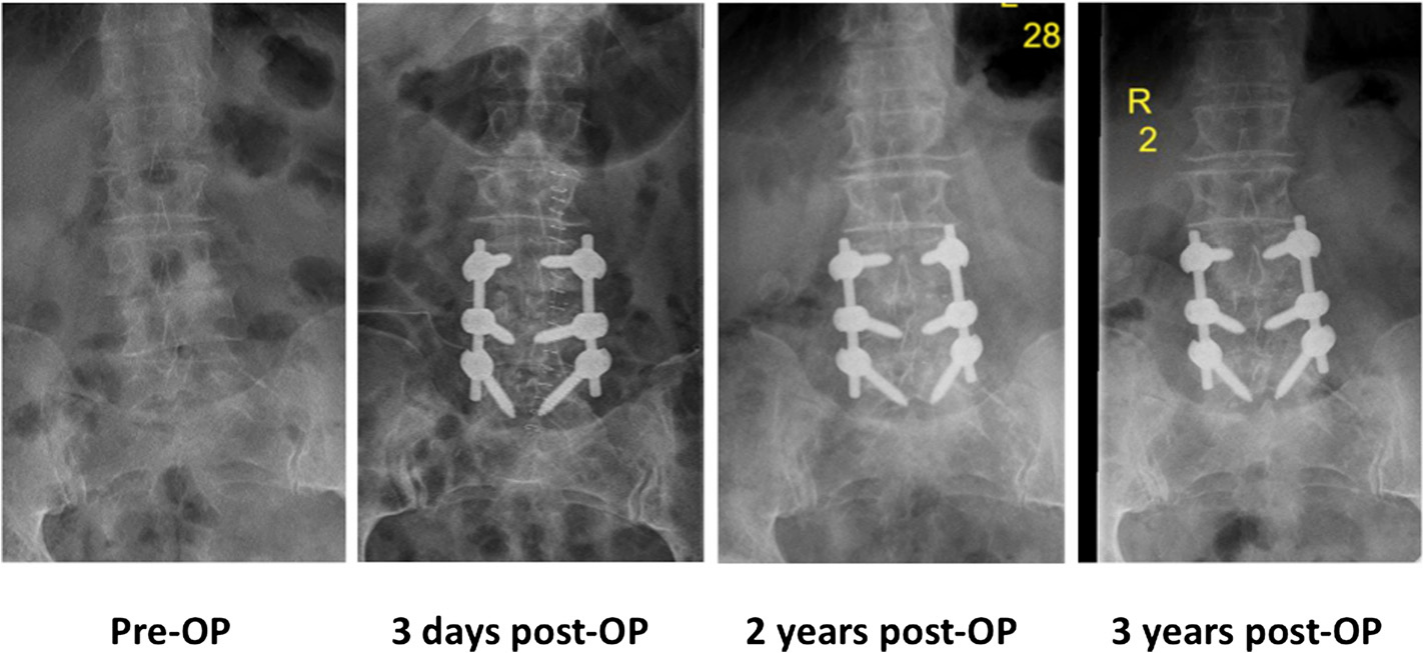
Pre- and post-operative conventional X-rays in the a.p. view (*upright standing*) throughout the 3 year follow-up

**Fig. 6:**
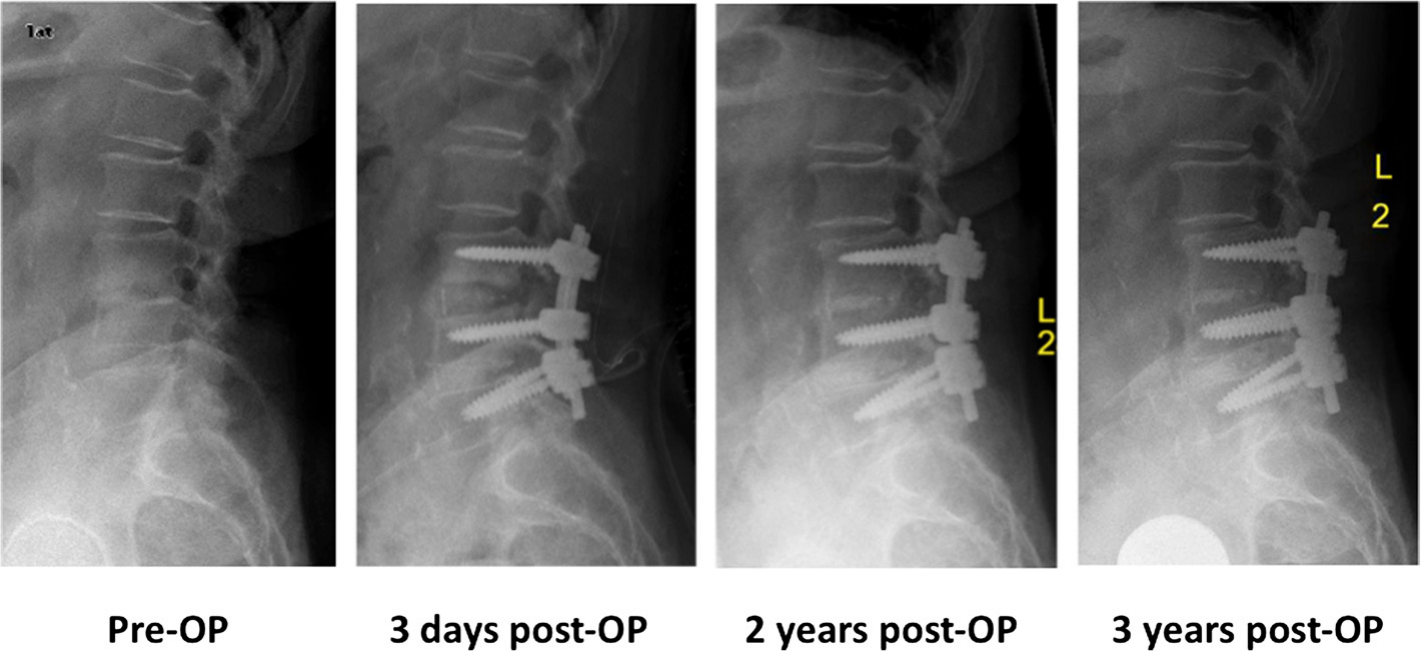
Pre- and postoperative conventional X-rays in the lateral view (*upright standing*) throughout the 3 year follow-up. In addition, the patient received a total hip arthroplasty of the left hip due to progressive hip arthritis, 2 years after her uneventful recovery from the lumbar spine surgery

From January 2009 through December 2013 a total number of 104 patients (62 male and 42 female patients) with a mean age of 75.0 ± 8.3 years presented with lumbar pyogenic spondylodiscitis requiring surgical therapy. Eighteen of these patients (4 male and 14 female patients, mean age 74.3 ± 7.2 years) were prospectively enrolled into the study by means of the above mentioned criteria (Table 1), and subsequently received the TLIF procedure with PEEK cage implantation as described. Upon admission, these 18 patients presented with an average VAS of 9.0 ± 0.5 and an ODI (%) of 77.0 ± 5.7. Laboratory results demonstrated mean baseline CRP serum level concentrations of 159.4 mg/l ± 53.9 mg/l and a mild leucocytosis with an average of 11.1/nl ± 4.8/nl. The most common comorbidity was a coronary heart disease with or without cardiomyopathy, respectively (Table 2). All 18 patients (*n* = 18) had a past medical history of acute bacterial infection involving either the nasopharynx or the respiratory tract but none of the patients presented with sepsis. Neurologic impairment was evident in two cases (*n* = 2), demonstrating lumbar radiculopathy with mild unilateral sensomotoric dysfunction. The average postoperative stay on the intensive care unit was 1.9 days (0–3 days). One patient (*n* = 1) demonstrated immobilizing pain (VAS 8/10) despite appropriate conservative treatment and an additional abscess formation within the right psoas muscle at the level of infection. The surgical procedure included drainage of the abscess via the ipslateral disc space by perforating the lateral anulus and carefully advancing a soft infant feeding tube into the respective area under fluorscopic guidance (Figs. 1, 2, 3). A second patient (*n* = 1) presented with a suspected bisegmental infection at the L3/4 and L4/5 level with secondary kyphosis and immobilizing pain (VAS 10/10). The surgical procedure consisted of a two level posterior decompression, removal of the purulent L3/4 disc, debridement of L4/5 and bisegemental fusion of L3-5 in TLIF technique (Figs. 4, 5, 6). However, only the L3/4 level resulted positive in the subsequent microbiological analysis.

**Table 1:** Indication for surgery (*n* patients)

Immobilizing pain	10
Failure of conservative therapy^a^	6
Instability with osseous lesions	5
Epidural/paravertebral abscess formation	4
Secondary kyphosis	2

^a^Patients diagnosed with lumbar spondylodiscitis treated conservatively for more than 6 weeks and/or demonstrating disease progression

**Table 2:** Co-morbidities (*n* patients)

Acute bacterial infection	18
Coronary heart disease/cardiomyopathy	16
Diabetes mellitus	12
Chronic obstructive pulmonary disease	4
Nephropathy	4

Overall, microbiological analyses of specimens obtained during the surgical debridement and lavage identified the pathogen in 13 cases (Table 3) with the most common being *Staphylococcus aureus*. Dual broad-spectrum i.v.-antibiotics (clindamycin and rifampicin) were continued for a 7-day minimum postoperatively. Thereafter, antibiotics were continued in adjustment with bacterial resistance and applied orally with a single drug antibiotic treatment (primarily clindamycin) by the time of discharge. In all cases, full ambulation was achieved within ten days after surgery and all patients discharged to recover in their domestic environment at an average of 16.0 ± 6.2 days postoperatively (hospitalization 10–26 days). The two patients with mild radiculopathy showed complete recovery and full ambulation at the 3 month clinical and radiographic follow-up without any signs of residual neurologic deficits (ASIA grade E). In addition, the average VAS decreased to 5.0 ± 1.8 with an average ODI (%) of 46.0 ± 14.3 with all inflammatory parameters back to normal levels, including full premorbid functional recovery at 3 months postoperatively.

**Table 3:** Pathogens detected (*n* patients)

Staphylococcus aureus	6
Staphylococcus epidermidis	4
Enterococcus faecalis	2
Escherichia coli	1
None detected	5

None of the 18 patients included in this study showed any signs of implant-related complications with bony fusion after 6 months post-operatively (exemplary Figs. 1, 2, 3). None of the cases were noted to have any signs of recurrent infection throughout the postoperative follow-up of up to 5 years.

## Discussion

Although the majority of patients with mild pyogenic spondylodiscitis showing no secondary bony destruction and instability are successfully treated nonsurgically, the appropriate management of cases meeting the criteria for a surgical intervention continues to be a controversial issue. Above all, patients demonstrating persistent or even immobilizing pain under conservative treatment remain highly challenging. Particularly in the lower lumbar region, sufficient immobilization via lumbar bracing by equally maintaining independent ambulation is often difficult (i.e. in obese patients) and may lead to significant issues in patient compliance. In addition to clinically evident and image verified disease progression, failure to respond to conservative therapy and/or uncontrollable pain have therefore been considered relative indications for surgical therapy [1, 16–18]. Furthermore, various discussions regarding the choice of approach and extent of surgical procedures have shown, at least in part, a strong dependence of the respective surgeon’s preference and expertise [9, 11, 19–21]. This becomes even more significant in cases where concomitant degenerative conditions within the affected segment must be equally addressed. Yet, the primary surgical aims and principles are clearly defined to eradicate the site of infection and stabilize the subsequent bony defect by a solid 360° interbody fusion with resistance to bacterial colonization [22]. In this context, we and others have since favoured the use of titanium cages over all autologous bone strut interpositioning due to the high rate of subsidence and osteonecrosis with subsequent loss of correction and the increased incidence of pseudarthrosis [3–5, 23, 24]. Furthermore, the use of iliac bone struts bears the risk of donor site morbidity and instability due to osteoporosis, particularly with respect to the majority of patients imperiled to pyogenic spondylodiscitis being elderly.

However, considering the asserted advantages of PEEK over metal implants with regard to cage subsidence, radiolucency and improved biomechanical behaviour (e.g. elastic modulus) [25], we sought to investigate the overall feasibility of implanting PEEK cages in the setting of pyogenic lumbar spondylodiscitis. In acknowledgment of a similar empirical trial reported by Pee and colleagues [11], we intended to investigate its practicality by means of a strictly posterior single-stage (TLIF) procedure. The fundamental hypothesis was to equally address all possible aspects of pyogenic lumbar spondylodiscitis (infected disc, bony destruction with subsequent instability and potential compromise of neural structures) in a single surgical approach and thus avoid the necessity of two separate interventions, including a repositioning of the patient. The efficacy of applying the TLIF-technique in degenerative disorders of the lumbar spine has been sufficiently proven [26–28]. A recent cadaver study by Rihn and colleagues demonstrated the open TLIF technique to allow more than 70 % of the total disc volume to be removed via an unilateral approach [29]. Similar results in vivo were reported by Javernick and colleagues [30]. Conferred to our approach in pyogenic lumbar spondylodiscitis, the TLIF technique equally enabled a sufficient removal of contaminated disc tissue and subsequent antiseptic irrigation. Furthermore, by concluding the procedure with careful irrigation of the exposed epidural space, the risk of dispersive contamination may be considered insignificant, respectively. Although our small patient cohort only represents a restricted selection of patients, the follow-up analyses of up to 5 years demonstrate a consistently promising outcome. Our results therefore correlate well with the observations reported by Pee and colleagues, and complement the overall results from recent small cohort analyses in cases of cervical spondylodiscitis [12, 13, 31]. In support of these clinical findings, a more recent in vitro investigation by Hahnel and colleagues was able to demonstrate PEEK surfaces to show equal or even lower grades of biofilm formation when compared with titanium implants.

In summary, we believe that minimizing operation time and surgical exposure by equally addressing all critical issues of the respective pathology may certainly benefit the overall outcome, particularly in patients with significant comorbidities. We hereby acknowledge, that this assertion is, at least in part, based on the comparison with other reports [11, 32], but also derives from our own unpublished observations in equivalent cases treated by single-stage anterior debridement and fusion combined with posterior screw-and-rod instrumentation.

Although today's literature still owes relevant data and long-term experience from larger cohorts on a high evidence based level to permit reliable guidelines, we believe that our data underlines two fundamental treatment principles in pyogenic lumbar spondylodiscitis:

1) a safe and radical debridement of the infectious site and 2) warranting segmental stability by reconstructing the physiological profile to achieve complete recovery and subsequently the best possible outcome.

Both clinical and radiological results of our study prove the use of PEEK interbody devices to be successful, thereby allowing a stable and solid bony fusion with sufficient decompression of neurological structures via the posterior TLIF approach. With minimally invasive surgical techniques gaining in importance, future studies using minimal access-TLIF techniques for pyogenic lumbar spondylodiscitis may promise equally beneficial outcomes. In addition, the future trend to generate an optimal bioactive implant-bone interface by surface modification (e.g. titanium, hydroxylapatite, β-TCP or calcium silicate composites) may also add to the advantages previously outlined by Rao and colleagues [33]. This includes the implementation of titanium coated PEEK interbody devices to reduce bacterial adherence and enhance osseointegration, respectively.

## Conclusion

Our results demonstrate that the single-step TLIF approach sufficiently addresses all aspects indicating the surgical treatment of pyogenic lumbar spondylodiscitis. Concomitantly, the use of an intervertebral oblique PEEK cage and autologous bone provide optimal stability via bridging of the bony endplates in cases where the majority of cortical bone is intact. In addition, this technique allows a minimally-invasive approach, which may further reduce iatrogenic morbidity in these high risk patients without the fear for recurrent infection. With today's advances in implant surface modification, the future use of titanium coated PEEK implants may significantly benefit the management of these detrimental conditions.

## References

[1] Akbar M, Sobottke R, Lehner B, Eichler M, Wang H, Carstens C (2012). Pyogenic spondylodiscitis: therapy algorithm and a new classification for therapeutic decision-making. Orthopade.

[2] Fantoni M, Trecarichi EM, Rossi B, Mazzotta V, Di Giacomo G, Nasto LA (2012). Epidemiological and clinical features of pyogenic spondylodiscitis. Eur Rev Med Pharmacol Sci..

[3] Korovessis P, Petsinis G, Koureas G, Iliopoulos P, Zacharatos S (2006). Anterior surgery with insertion of titanium mesh cage and posterior instrumented fusion performed sequentially on the same day under one anesthesia for septic spondylitis of thoracolumbar spine: is the use of titanium mesh cages safe?. Spine (Phila Pa 1976).

[4] Robinson Y, Tschoeke SK, Finke T, Kayser R, Ertel W, Heyde CE (2008). Successful treatment of spondylodiscitis using titanium cages: a 3-year follow-up of 22 consecutive patients. Acta Orthop.

[5] Robinson Y, Tschoeke SK, Kayser R, Boehm H, Heyde CE (2009). Reconstruction of large defects in vertebral osteomyelitis with expandable titanium cages. Int Orthop.

[6] Ruf M, Stoltze D, Merk HR, Ames M, Harms J (2007). Treatment of vertebral osteomyelitis by radical debridement and stabilization using titanium mesh cages. Spine (Phila Pa 1976).

[7] Sasso RC, LeHuec JC, Shaffrey C, Spine Interbody Research G (2005). Iliac crest bone graft donor site pain after anterior lumbar interbody fusion: a prospective patient satisfaction outcome assessment. J Spinal Disord Tech.

[8] Endres S, Wilke A (2012). Posterior interbody grafting and instrumentation for spondylodiscitis. J Orthop Surg (Hong Kong)..

[9] Ha KY, Shin JH, Kim KW, Na KH (2007). The fate of anterior autogenous bone graft after anterior radical surgery with or without posterior instrumentation in the treatment of pyogenic lumbar spondylodiscitis. Spine (Phila Pa 1976).

[10] Lerner T, Hackenberg L, Rosler S, Joosten U, Halm H, Liljenqvist U (2005). Surgical therapy of unspecific and specific Spondylodiscitis. Z Orthop Ihre Grenzgeb.

[11] Pee YH, Park JD, Choi YG, Lee SH (2008). Anterior debridement and fusion followed by posterior pedicle screw fixation in pyogenic spondylodiscitis: autologous iliac bone strut versus cage. J Neurosurg Spine.

[12] Brase A, Ringel F, Stuer C, Meyer B, Stoffel M (2010). Debridement and fusion with polyetheretherketone implants in purulent spondylodiscitis: a clinical experience with nine patients. Acta Neurochir (Wien).

[13] Mondorf Y, Gaab MR, Oertel JM (2009). PEEK cage cervical ventral fusion in spondylodiscitis. Acta Neurochir (Wien).

[14] Marshall JC (2000). SIRS and MODS: what is their relevance to the science and practice of intensive care?. Shock.

[15] Calandra T, Cohen J (2005). International Sepsis Forum Definition of Infection in the ICUCC. The international sepsis forum consensus conference on definitions of infection in the intensive care unit. Crit Care Med.

[16] Butler JS, Shelly MJ, Timlin M, Powderly WG, O'Byrne JM (2006). Nontuberculous pyogenic spinal infection in adults: a 12-year experience from a tertiary referral center. Spine (Phila Pa 1976).

[17] Duarte RM, Vaccaro AR (2013). Spinal infection: state of the art and management algorithm. Eur Spine J.

[18] Zarghooni K, Rollinghoff M, Sobottke R, Eysel P (2012). Treatment of spondylodiscitis. Int Orthop.

[19] Klockner C, Valencia R (2003). Sagittal alignment after anterior debridement and fusion with or without additional posterior instrumentation in the treatment of pyogenic and tuberculous spondylodiscitis. Spine (Phila Pa 1976).

[20] Linhardt O, Kruger A, Krodel A (2004). First results of anterior versus posterior instrumentation-fusion in the treatment of spondylodiscitis. Z Orthop Ihre Grenzgeb.

[21] Mann S, Schutze M, Sola S, Piek J (2004). Nonspecific pyogenic spondylodiscitis: clinical manifestations, surgical treatment, and outcome in 24 patients. Neurosurg Focus.

[22] Flierl MA, Beauchamp KM, Bolles GE, Moore EE, Stahel PF (2009). Fatal outcome after insufficient spine fixation for pyogenic thoracic spondylodiscitis: an imperative for 360 degrees fusion of the infected spine. Patient Saf Surg.

[23] Fayazi AH, Ludwig SC, Dabbah M, Bryan Butler R, Gelb DE (2004). Preliminary results of staged anterior debridement and reconstruction using titanium mesh cages in the treatment of thoracolumbar vertebral osteomyelitis. Spine J.

[24] Kuklo TR, Potter BK, Bell RS, Moquin RR, Rosner MK (2006). Single-stage treatment of pyogenic spinal infection with titanium mesh cages. J Spinal Disord Tech.

[25] Kurtz SM, Devine JN (2007). PEEK biomaterials in trauma, orthopedic, and spinal implants. Biomaterials.

[26] Harris BM, Hilibrand AS, Savas PE, Pellegrino A, Vaccaro AR, Siegler S (2004). Transforaminal lumbar interbody fusion: the effect of various instrumentation techniques on the flexibility of the lumbar spine. Spine (Phila Pa 1976).

[27] Salehi SA, Tawk R, Ganju A, LaMarca F, Liu JC, Ondra SL (2004). Transforaminal lumbar interbody fusion: surgical technique and results in 24 patients. Neurosurgery.

[28] Zhao J, Hou T, Wang X, Ma S (2003). Posterior lumbar interbody fusion using one diagonal fusion cage with transpedicular screw/rod fixation. Eur Spine J.

[29] Rihn JA, Gandhi SD, Sheehan P, Vaccaro AR, Hilibrand AS, Albert TJ (2014). Disc space preparation in transforaminal lumbar interbody fusion: a comparison of minimally invasive and open approaches. Clin Orthop Relat Res.

[30] Javernick MA, Kuklo TR, Polly DW (2003). Transforaminal lumbar interbody fusion: unilateral versus bilateral disk removal--an in vivo study. Am J Orthop (Belle Mead NJ)..

[31] Walter J, Kuhn SA, Reichart R, Kalff R, Ewald C (2010). PEEK cages as a potential alternative in the treatment of cervical spondylodiscitis: a preliminary report on a patient series. Eur Spine J.

[32] Eysel P, Hopf C, Vogel I, Rompe JD (1997). Primary stable anterior instrumentation or dorsoventral spondylodesis in spondylodiscitis? Results of a comparative study. Eur Spine J.

[33] Rao PJ, Pelletier MH, Walsh WR, Mobbs RJ (2014). Spine interbody implants: material selection and modification, functionalization and bioactivation of surfaces to improve osseointegration. Orthop Surg.

